# Effects of Curcumin and Lactoferrin to Inhibit the Growth and Migration of Prostatic Cancer Cells

**DOI:** 10.3390/ijerph192316193

**Published:** 2022-12-03

**Authors:** Erica Costantini, Marta Di Nicola, Michele Marchioni, Lisa Aielli, Marcella Reale, Luigi Schips

**Affiliations:** 1 Department of Medicine and Aging Sciences, University “G. d’Annunzio”, Via dei Vestini, 66100 Chieti, Italy; 2 Department of Medical, Oral and Biotechnological Sciences, University “G. d’Annunzio”, Via dei Vestini, 66100 Chieti, Italy; marta.dinicola@unich.it (M.D.N.); luigi.schips@unich.it (L.S.); 3 Department of Innovative Technologies in Medicine and Dentistry, University “G. d’Annunzio”, Via dei Vestini, 66100 Chieti, Italy; lisa.aielli@unich.it (L.A.); mreale@unich.it (M.R.)

**Keywords:** prostate cancer, inflammation, curcumin, lactoferrin

## Abstract

Prostate cancer remains one of the main causes of death for men worldwide. Despite recent advances in cancer treatment, patients develop resistance after an initial period of optimal efficacy. Nowadays, it is accepted that natural compounds can result in health benefits with a preventive or adjuvant effect. The purpose of this study was to evaluate the effects of curcumin (CU), a bioactive compound in the spice turmeric, and lactoferrin (LF), a natural glycoprotein with immunomodulatory properties, on DU145 and PC3. Prostate cancer cells were cultured with and without LF (175 μM) and CU (2.5 μg/mL and 5 μg/mL), alone and in combination. Cell viability, migration ability, death receptors (DRs), and integrins (α3, β1) gene expression were evaluated, as well as human annexin V quantification and Akt phosphorylation. Differences among cells group, defined according to the treatment used, were assessed with ANOVA. The results showed that the effects of CU and LF are different between the two prostatic cell lines analyzed. In DU145, a reduction in cell proliferation and migration is reported both in the presence of single and combined treatments. In PC3 cells, there is a significant reduction in proliferation in the presence of CU alone, while the inhibition of migration is mainly related to the LF treatment and its combination with CU, compared to untreated cells. Moreover, the reduction in gene expression of integrins and Akt pathway activation were observed mostly in the presence of the CU and LF combination, including the upregulation of DR and annexin V levels, with greater significance for the DU145 cells. In conclusion, our results suggest that CU and LF may have a potentially beneficial effect, mainly when administered in combination, leading to a reduction in cancer cells’ aggressiveness.

## 1. Introduction

Prostate cancer represents one of the most common genitourinary malignancies in males according to the epidemiology statistics of global cancer from the World Health Organization, which report more than 1.41 million cases, with morbidity ranked 4th in 2020 [1,2]. Patients affected by localized prostate tumors might expect 5-year survival probability >95%, but in the 35% of patients with advanced disease or with metastatic lesions, this survival probability turns out to be reduced [3].

Significant advances in treatment modalities and the consistent overall survival advantage resulting from the use of new therapies derive from the application of systemic treatments, based on the use of androgen deprivation treatments, in association with new androgen-targeted therapies (ARATs), such as apalutamide, darolutamide, enzalutamide and/or abiraterone acetate, or chemotherapy, as docetaxel [4].

Despite this improvement in prostate cancer treatment, there is a huge demand for the development of alternative treatments for prostate cancer patients’ health and the improvement of their quality of life [5].

The application of medicinal herbs for health and wellness, by natural products (or rather their beneficial bioactive compounds with low toxicity), is spread all over the world. To date, hundreds of molecules have been originally obtained from these natural products and identified as potential health modifiers [6,7]. In recent times, it has been observed across the world that some natural products exhibit great potential with respect to sensitizing cancer cells and reducing their malignancy grade, although the clinical potential remains unsatisfactory [8,9,10]. Curcumin (CU), the main curcuminoid of the Indian spice turmeric, is extracted from the turmeric root. CU is a small natural hydrophobic polyphenol characterized by two aromatic rings connected by a seven-carbon linker, which interacts with multiple cellular activities [11]. CU is metabolized in different conjugates, with the final production of glucuronide and sulfate. The latter CU conjugates are involved in the main metabolic pathways and act on multiple intracellular targets, thanks to their lipophilic nature [12]. In vitro and in vivo studies have shown numerous benefits from CU administration. Anti-inflammatory, antioxidant, antibacterial, anti-fungal, antiviral, and anti-cancer properties for the treatment of several pathological conditions have all been reported in clinical trials [13,14,15,16].

The limits of CU application are related to the lower bioavailability and the extensive metabolism of this polyphenol, together with the need to use quantities greater than 10 μM to obtain a notable result [17]. Despite continuous research innovations, such as associating it with compounds capable of increasing biodistribution (liposomes, chitosan, and solid-lipid microparticles), these drawbacks have not yet been overcome, requiring the development and evaluation of new strategies [12,18,19,20]. To overcome these application limits, we suggest the evaluation of a combination of low-dose CU in the presence of lactoferrin (LF).

Notably, LF is a natural iron-binding glycoprotein with a high ability to enter the cell nucleus, both through binding of specific receptors and/or spontaneous cellular uptake [21]. LF presents a well-conserved, monomeric 80 kDa single polypeptide chain glycoprotein of about 690 amino acid residues, and is considered a first-line defense protein involved in protecting against a multitude of microbial infections and in the prevention of systemic inflammation [22]. LF shows multiple biological functions, such as immunoregulatory, anti-inflammatory, and anti-viral abilities, as well as the ability to act as a tumor suppressor through the oxidant system and cell cycle regulation. The high bioavailability of LF, and the high selectivity toward tumor cells and molecular targets that control tumor proliferation, migration, invasion, and metastasis, make LF an ideal vector for cancer prevention and treatment [23,24]. Therefore, the exploration of the combination of natural compounds may provide alternatives to support the treatment of prostate cancer. Combining CU and LF can help strengthen the immune system, prevent the disease from progressing to the severe stage, and suppress the degree of inflammation. In line with the latest research and taking in mind this background, we aimed to evaluate the effects of LF and CU in two prostatic cancer cells, PC3 and DU145, with different metastatic potential [25] and which are resistant to many chemotherapy drugs and apoptosis inducers [26].

## 2. Materials and Methods

### 2.1. Reagents

**Figure 1:**
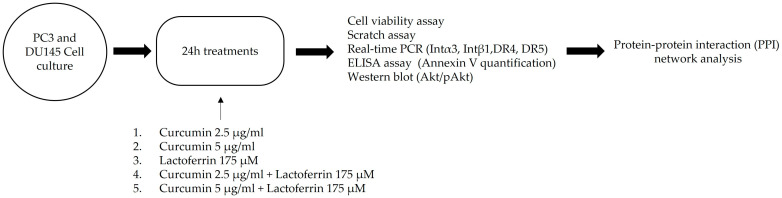
Graphical scheme of study.

Lactoferrin (L4894, Sigma-Aldrich, St. Louis, MO, USA), curcumin (C1386, Sigma-Aldrich), and 3-(4,5-dimethylthiazol-2-yl)-2,5-diphenyltetrazolium bromide (MTT, Sigma-Aldrich) were purchased from Merck (St. Louis, MO, USA). Based on previous dose-response experiments, we chose to evaluate LF at 175 μM, and CU at 2.5 μg/mL and 5 μg/mL, for the subsequent experiments (Figure 1).

### 2.2. Cells

Human PC cell lines (DU145, PC3) were cultured in Dulbecco’s modified Eagle’s medium/1640 supplemented with 10% fetal bovine serum (Sigma) and 1% penicillin-streptomycin, at 37 °C, in humidified air containing 5% CO_2_.

### 2.3. Cell Viability Assay

The ability of LF (175 μM) and CU (2.5 μg/mL and 5 μg/mL) to affect PC3 and DU145 cell viability was determined using a standard colorimetric MTT reduction assay. Cells in exponential growth were harvested by trypsinization and seeded at a concentration of approximately 0.8 × 10^5^ cells per 100 μL per well into 96-well plates. After reaching confluence, the medium was removed and a fresh medium with defined concentrations of LF and CU, alone or in combination, was added to the cultures in parallel. Control cells without treatments were cultured using the same conditions. After treatment for 24 h, the medium was removed and replaced by a fresh drug-free medium (100 μL/well), and 10 μL of MTT solution (5 mg/mL) was added to each well. Cells were incubated for 3 h at 37 °C, and the supernatants were carefully removed. Then, 100 μL of dimethyl sulfoxide (DMSO, Merck, St. Louis, MO, USA) was added to each well to dissolve the crystal products. Absorbances were measured at 550 nm using a Glomax Multireader spectrophotometer (Promega, Madison, WI, USA).

### 2.4. Scratch Assay

DU145 and PC3 cells were seeded into 6-well plates at a concentration of 1.2 × 10^6^ cells/well in 1 mL of complete growth medium. When the cell density reached above 90%, a scratch wound was made by scraping the cell layer using a p10 pipette tip. Later, the cells were washed with phosphate buffered saline (PBS) (Merck, St. Louis, MO, USA) to remove cell debris, and the wounded cultures were incubated in a fresh growth medium with LF (175 μM) and CU (2.5 μg/mL and 5 μg/mL), alone or in combination. At 6 and 24 h after scratching, pictures were taken of each wound, using a digital camera coupled to an inverted microscope (Leica Microsystem, Milan, Italy).) to investigate collective cell migration [27]. In each case, the images were captured using a reference grid, in order to photograph the same fields of wells. The wound closure rate was determined by measuring in centimeters (cm) the distance between the edges of the wound. The experiments were performed in triplicate.

### 2.5. RNA Extraction, Purification, Retro-Transcription, and Real-Time PCR Analysis

Quantitative real-time PCR (qPCR) was performed on a Biorad CFX96 (Bio-Rad, Hercules, CA, USA) using the GoTaq^®^ qPCR kit (Promega, Madison, WI, USA), in addition to the forward and reverse primers for each gene. The nucleic acid sequences of the primers were as follows: Integrin (INT)β1: Fw: 5′-ATCCCTGAAAGTCCCAAGTG-3′, Rw: 5′-ACGCACTCTCCATTGTTACTG-3′; INTα3: Fw: 5′-ATGTGGCTTGGAGTGACTG-3′, Rw: 5′-CATCTCGTTGTGGTAGGTCTG-3′; Death Receptor (DR)4: Fw: 5′-GATTACACCAACGCTTCCAAC-3′, Rw: 5′-CTACACTTCCGGCACATCTC-3′; DR5: Fw: 5′-ACCACGACCAGAAACACAG-3′, Rw: 5′-AAGACTACGGCTGCAACTG-3′; Glyceraldehyde-3-phosphate dehydrogenase (GAPDH): Fw: 5′-ATGGCTATGATGGAGGTCCAG-3′, Rw: 5′-TTGTCCTGCATCTGCTTCAGC-3′. Amplification and cycle were run at 95 °C for 15 min for the initial activation, followed by denaturation at 95 °C for 30 s, annealing at 55 °C for 30 s, and elongation at 72 °C for 30 s, repeated for 40 cycles. Melting curves were performed after qPCR to demonstrate the specific amplification of single products of interest. A standard curve assay was performed to determine the amplification efficiency of the primers used. Relative fold changes in the expression of target genes were determined using the comparative 2^−ΔΔCt^ with the β-actin gene as an internal control to normalize the level of target gene expression. ΔΔCT represents the difference between the mean ΔCT_(treatment group)_ and mean ΔCT_(control group)_, where ΔCT is the difference between the mean CT gene of interest and the mean CT of the internal control gene in each sample [28].

### 2.6. Annexin V Detection

Human annexin V ELISA assays (Affymetrix eBioscience, Thermo Fisher Scientific, Waltham, MA, USA) were used for the quantitative detection of annexin V in the cell culture supernatant, to evaluate the influence of LF (175 μM) and CU (2.5 μg/mL and 5 μg/mL), alone or in combination, in PC3 and DU145 cells.

After 24 h of treatments, supernatant samples were collected and frozen at −20 °C to avoid loss of bioactive human annexin V, following the assay recommendations.

Before testing, samples were brought to room temperature and centrifugated to eliminate precipitates, and 50 μL of each sample supernatant was plated on an anti-human-annexin-V-antibody-coated well, in duplicate, and processed following manufacturer’s instructions. The absorbance of each microwell was assessed on a spectrophotometer using 450 nm as the wavelength, and the concentration of circulating human annexin V was calculated with the standard curve (ng/mL).

### 2.7. Western Blot

PC3 and DU145 cells (1.2 × 10^6^ cells/well) were seeded in a 6-well plate. After reaching 80% confluence, cells were incubated with LF (175 μM) and CU (2.5 μg/mL and 5 μg/mL), alone or in combination, for 24 h. After the treatment, cells were lysed using RIPA buffer (Merck, St. Louis, MO, USA) and added to the protease inhibitor cocktail (Merck St. Louis, MO, USA) and the phosphatase inhibitor (Sigma Aldrich) for 30 min on ice. After centrifugation at 10,000× *g*, a bicinchoninic acid (BCA) protein assay kit (Thermo Fisher Scientific, Waltham, MA, USA) was used to determine the protein concentration. The protein samples (35 μg) were separated in 10% sodium dodecyl sulfate (SDS) polyacrylamide gels and transferred to a polyvinylidene difluoride (PVDF) membrane (Bio-Rad Inc., Hercules, CA, USA). The membrane was blocked with 5% skim milk in Tris-buffered saline with 0.1% Tween-20 (TBST) for 1 h at room temperature and then incubated overnight at 4 °C with the specific primary antibodies, Akt (Protein kinase B, PKB, dil. 1:500) and phospho-Akt (Ser473, dil. 1:500). The membranes were washed in TBST three times and incubated with horseradish peroxidase (HRP)-conjugated secondary antibody (dil. 1:10,000) at room temperature for 2 h. Protein bands were visualized using a chemiluminescence detection kit (Euroclone, MI, Italy), scanning the membrane with a ChemiDoc XRS (Bio-Rad Inc., Hercules, CA, USA). β-actin (dil. 1:500) was used as the loading control.

### 2.8. Statistical Analysis

Descriptive statistics relied on mean and standard deviation (±SD) for continuous variables, and frequencies and percentages (%) for categorical variables. We relied on ANOVA for assessing differences in mean across groups defined according to the treatment. Post-hoc analyses tested differences with the reference group, namely “untreated”. False discovery rate (FDR) was used for multiple testing corrections. Protein expression was tested compared to one by mean of Student’s *t*-test. Box and whisker plots graphically represented median and interquartile ranges with minimum and maximum values without outliers. Bar plots graphically depicted means and standard deviations. All statistical tests were two-sided. The level of significance was set at *p* < 0.05. Analyses were performed using the R software environment for statistical computing and graphics (version 4.1.2; http://www.r-project.org/ (accessed on 20 July 2022)).

### 2.9. Protein-Protein Interaction (PPI) Network Analysis

The search tool for retrieval of interacting genes (STRING) (https://string-db.org (accessed on 12 September 2022)) database, which integrates both known and predicted PPIs, was applied to predict functional interactions between proteins [29]. To assess potential interactions between expressed genes, the STRING tool was employed. Active interaction sources, including text mining, experiments, databases, and co-expression, as well as species limited to “Homo sapiens” and an interaction score > 0.4 were applied to construct the PPI networks.

## 3. Results

### 3.1. Effects of Curcumin and Lactoferrin on Cell Growth

**Figure 2:**
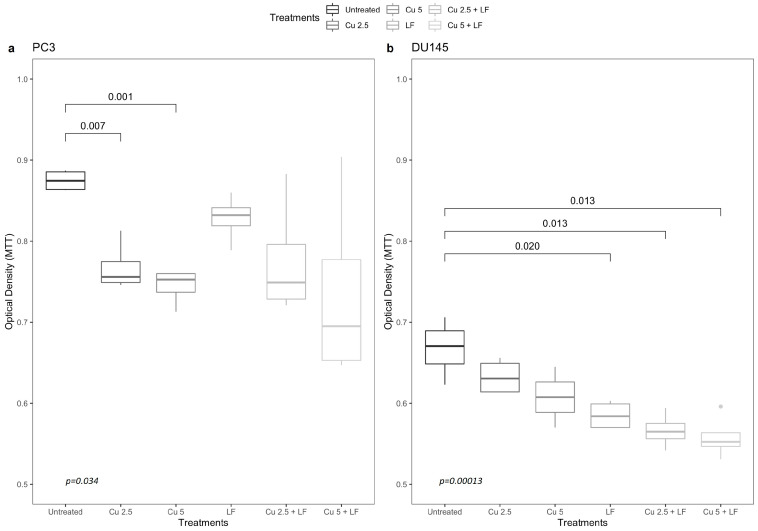
The inhibitory effect of CU and LF on the proliferation of (**a**) PC3 and (**b**) DU145 cells. Prostatic cells were treated with CU (2.5 μg/mL; 5 μg/mL) and LF (175 μM) for 24 h. The optical density values (490 nm) for cell viability were measured by MTT assay using DMSO as a positive control. Each assay was performed in triplicate. Box and whisker plots show 25th and 75th percentile range (box) with 95% confidence interval (whiskers) and median values (transverse lines in boxes).

The interference ability of CU and LF with androgen cancer-promoting activity was assessed firstly by examining their effect on cancer cell proliferation. To this end, we used the human prostate cancer cell lines PC3 and DU145, which are androgen resistant. The treatments with CU 2.5 μg/mL (*p* = 0.007) and 5 μg/mL (*p* = 0.001) reduced the proliferation rate of PC3 cells in a dose-dependent manner (Figure 2a). Treatments with LF 175 μM, alone or in association with CU, resulted in decreased cell proliferation compared to untreated cells, although not significantly.

In DU145 prostate cells, growth was not significantly reduced by CU in a dose-dependent manner. In contrast, LF 175 μM significantly reduced cell growth compared to untreated cells (*p* = 0.020). Additionally, the combination of LF with CU, at both concentrations (CU 2.5 + LF and CU 5 + LF), reduced cell growth in a highly significant way (*p* = 0.013) (Figure 2b). Thus, the antiproliferative effect of CU and LF seems to be cell-type-dependent.

### 3.2. Migration Assay

**Figure 3:**
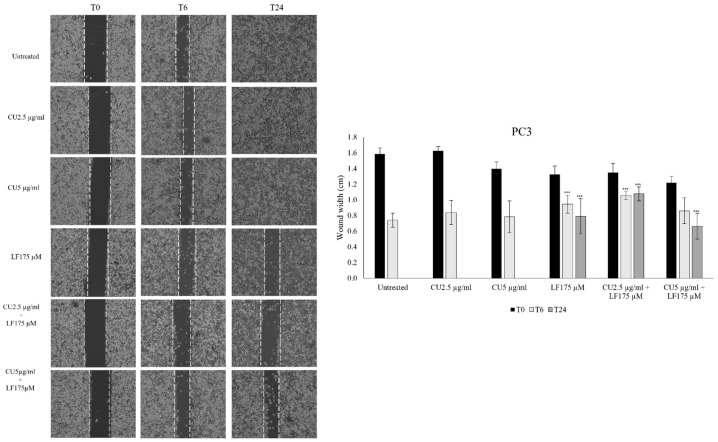
PC3 cell migration assay. Images from a scratch assay experiment at different time points. PC3 cells were wounded with a p10 pipette tip, incubated with CU 2.5 μg/mL, CU 5 μg/mL, and LF 175 μM, alone and in association, and imaged after 6 and 24 h using a microscope equipped with a photo camera. Scale bar = 120 μm. Six random views were chosen along the scratch wound in each well at ×100. The experiments were performed in triplicate and expressed as mean ± SD. *** *p* < 0.001 in comparison with untreated cells at the same time point.

To evaluate the impact of CU and LF on cell migration, the wound-healing assay was performed. After 6 h, PC3 cell migration was significantly inhibited by LF 175 μM alone (*p* = 0.010), and by CU 2.5 μg/mL in combination with LF 175 μM (*p* < 0.001), as compared to untreated PC3 cells at the same time point. After 24 h, the significantly reduced migration of PC3 cells was observed in the presence of LF 175 μM alone, and in combination with both CU 2.5 μg/mL (*p* < 0.001) and CU 5 μg/mL (*p* < 0.001), as compared to untreated cells at 24 h (Figure 3).

**Figure 4:**
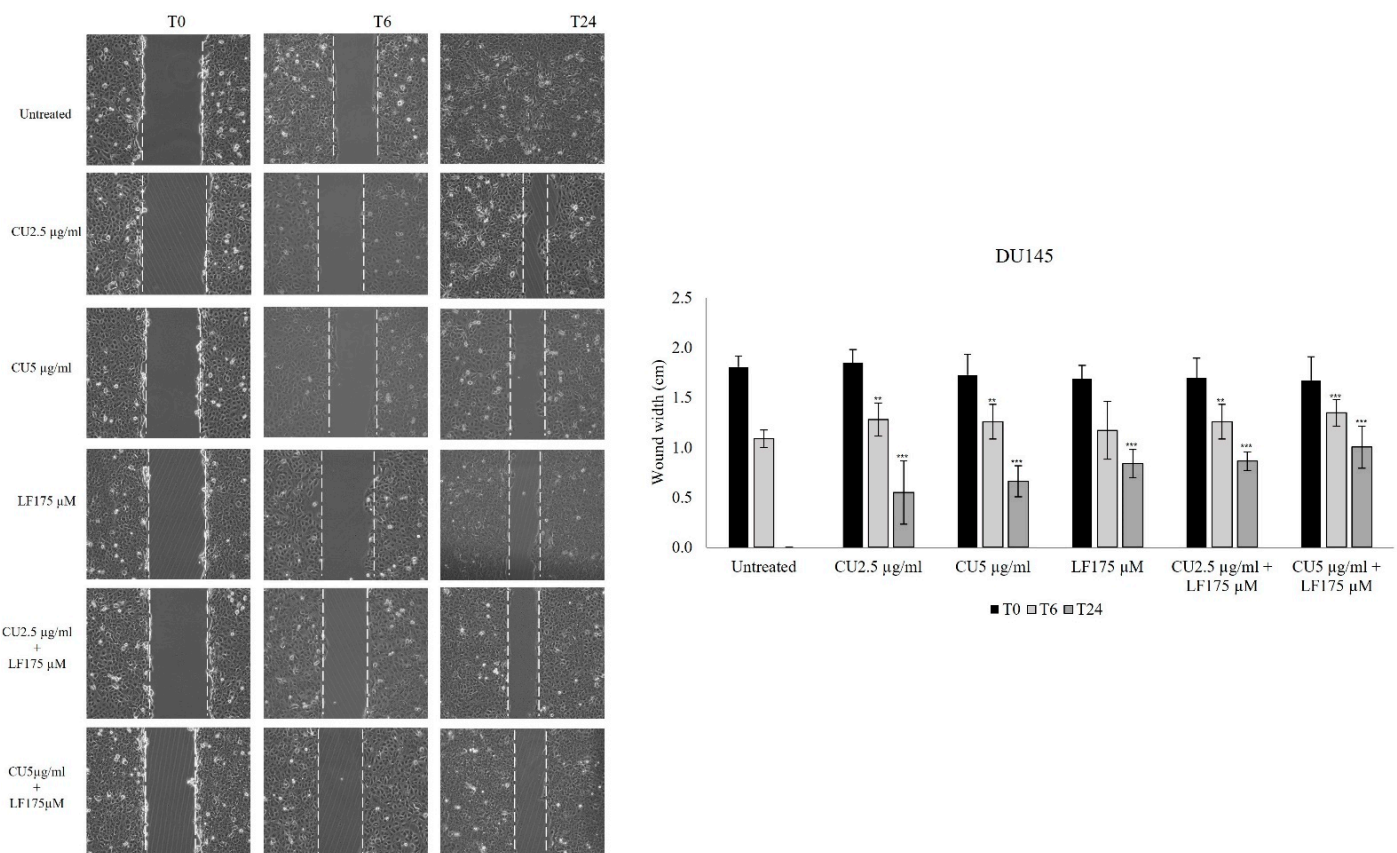
DU145 cell migration assay. Images from a scratch assay experiment at different time points. DU145 cells were wounded with a p10 pipette tip, incubated with CU 2.5 μg/mL, CU 5 μg/mL, and LF 175 μM, alone and in association, and imaged after 6 and 24 h using a microscope equipped with a photo camera. Scale bar = 120 μm. Six random views were chosen along the scratch wound in each well by microscopic magnification ×100. The experiments were performed in triplicate and expressed as mean ± SD. ** *p* < 0.01; *** *p* < 0.001 in comparison with untreated cells at the same time point.

A scratch assay was also applied to evaluate the effect of CU and LF on DU145 migration. The results showed that after 6 h, CU 2.5 μg/mL, CU 5 μg/mL, and their combinations with LF 175 μM, significantly inhibited cell migration in comparison to untreated cells (*p* < 0.05). By contrast, LF alone did not significantly reduce the width of the wound after 6 h (Figure 4).

### 3.3. Effect of CU 2.5 μg/mL, CU 5 μg/mL, and LF 175 μM on Integrin Gene Expression

**Figure 5:**
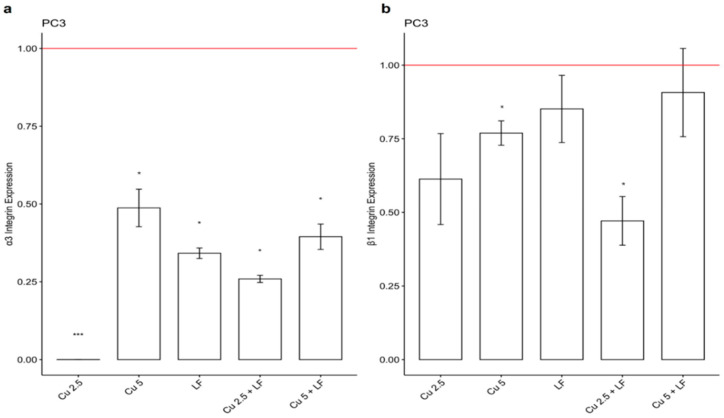
Integrin α3 (**a**) and β1 (**b**) gene expression levels. Effects of CU 2.5 μg/mL, CU 5 μg/mL, and LF 175 μM, alone and in association, on integrin α3 and β1 mRNA expression in PC3 cells. Fold change, calculated by qPCR, is reported as gene expression levels (2^−ΔΔCt^) in treated PC3 cells and compared to untreated cells, assumed as 1. * *p* < 0.05; *** *p* < 0.001. Data are expressed as mean ± SD.

To better evaluate the human prostate cancer cell line invasiveness, we analyzed the expression of α3 and β1 integrin involved in tumor progression. In addition to the reduction in cell migration, in our work, PC3 showed a significant reduction in the expression of α3 in the presence of CU 2.5 μg/mL (*p* < 0.001), CU 5 μg/mL, LF 175 μM (*p* < 0.05), CU 2.5 + LF (*p* < 0.05), and CU 5 + LF (*p* < 0.05) (Figure 5a). Additionally, for integrin β1, a decreased gene expression was observed in comparison with untreated cells, although in a not significant way (Figure 5b).

**Figure 6:**
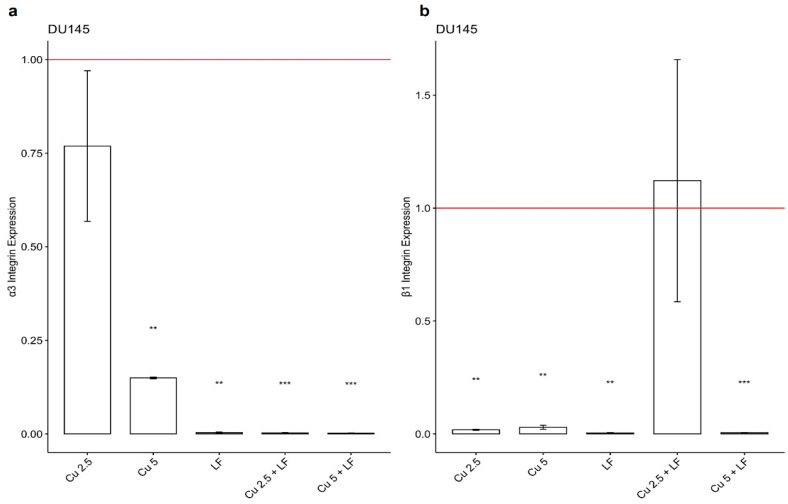
Integrin α3 (**a**) and β1 (**b**) gene expression levels. Effects of CU 2.5 μg/mL, CU 5 μg/mL, and LF 175 μM, alone and in association, on integrin α3 and β1 mRNA in DU145 cells. Fold change, calculated by qPCR, is reported as gene expression levels (2^−ΔΔCt^) in treated DU145 cells and compared to untreated cells, assumed as 1. ** *p* < 0.01; *** *p* < 0.001. Data are expressed as mean ± SD.

In DU145, a significant reduction in the expression levels of integrin α3 and β1 was observed in the presence of CU 2.5 μg/mL, CU 5 μg/mL, and LF 175 μM, alone and in association, compared to untreated cells. Our data clearly showed that treatment with CU and LF both significantly reduced the α3 gene expression, mostly pronounced in the presence of LF 175 μM (Figure 6a).

Additionally, a significant reduction was observed for the integrin β1 subunit (Figure 6b), in the presence of the CU and LF treatments, alone and in combination. When DU145 cells were treated with CU 2.5 + LF, gene expression was similar to that of the untreated group, suggesting that the α3 and β1 integrin subunits differently regulate the growth and invasion of tumor cells in a context-dependent manner.

### 3.4. Effect of CU 2.5 μg/mL, CU 5 μg/mL, and LF 175 μM on DR4 and DR5 Gene Expression

**Figure 7:**
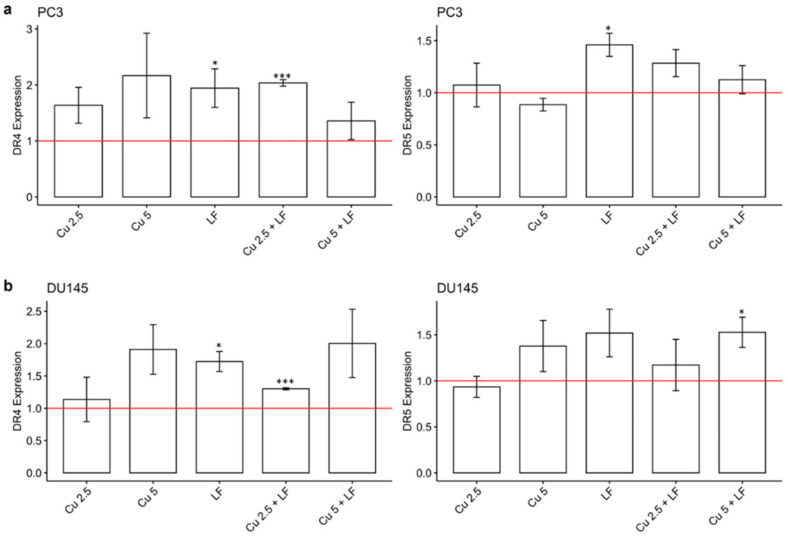
DR4 and DR5 gene expression levels. Effects of CU 2.5 μg/mL, CU 5 μg/mL, and LF 175 μM, alone and in association, on DR4 and DR5 mRNA in PC3 (**a**) and DU145 (**b**) cells. Fold change, calculated by qPCR, is reported as gene expression levels (2^−ΔΔCt^) in treated cells and compared to untreated cells, assumed as 1 (reported as red-lines). * *p* < 0.05; *** *p* < 0.001. Data are expressed as mean ± SD.

The DR4 and DR5 are expressed on the cell surface and are activated by binding with tumor-necrosis-factor-related apoptosis-inducing ligands (TRAILs). Changes in the gene expression levels of the two DRs may influence apoptosis signaling. Since their expression is mostly related to the cancer environment, we investigated the ability of CU and LF, alone or in association, to regulate DR4- and DR5-induced apoptosis. As shown in Figure 7, in both PC3 and DU145 prostate cells, we observed a significant increase in DR4 gene expression in cells treated with LF compared to untreated cells; this significant increase was induced by treatment with the combination of CU 2.5 μg/mL and LF. DR5 gene expression levels were significantly increased by LF treatment (in PC3 *p* < 0.05), and also by CU 2.5 μg/mL and LF (although not significantly).

### 3.5. Effect of Treatments on annexin V

To investigate the effect of CU and LF, alone or in combination, on apoptosis, the total extracellular amount of annexin V was evaluated in the cell culture supernatant collected after 24 h of treatment with CU 2.5 μg/mL, CU 5 μg/mL, and LF 175 μM.

**Figure 8:**
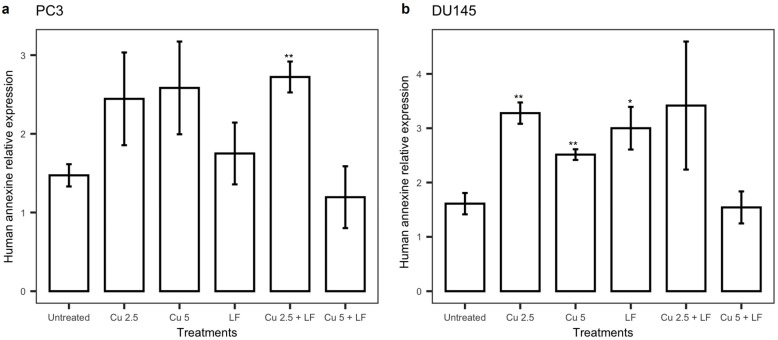
Annexin V levels. The annexin V levels, in PC3 (**a**) and DU145 (**b**), were determined using the immunoenzymatic assay. Data were reported as the mean ± SD of ng/mL, assessed for each sample in triplicate. Data are expressed as mean ± SD. * *p* < 0.05; ** *p* < 0.01.

In PC3 cells treated with CU 2.5 μg/mL and CU 5 μg/mL, an increase in human annexin V concentrations was observed relative to untreated cells, although not significantly; additionally, a smaller increase was detected after LF treatment as compared to untreated cells. Thus, we then evaluated whether the combination of CU and LF would differently modulate the release of annexin V. Our results showed a significant increase (*p* = 0.012) after CU 2.5 μg/mL + LF treatment, while the combination of CU 5 μg/mL + LF did not affect the annexin V levels (Figure 8a).

Incubation of DU145 in the presence of CU 2.5 μg/mL, CU 5 μg/mL, and LF 175 μM induced a significant increase in annexin V levels, suggesting their pro-apoptotic activity, in accord with reduced cell growth. Moreover, an increase in the annexin V levels was observed when CU 2.5 μg/mL was used as a combined treatment with LF, although not significantly (Figure 8b).

### 3.6. Effects of Treatments on Akt Pathway

**Figure 9:**
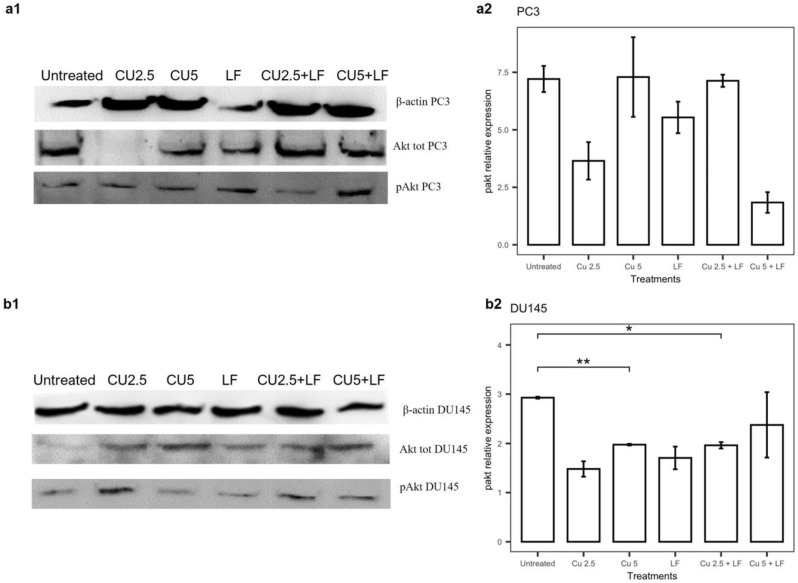
Effect of CU 2.5 μg/mL, CU 5 μg/mL, and LF 175 μM, alone and in association, on Akt phosphorylation in PC3 (**a**) and DU145 (**b**) cells. Protein expression of Akt, pAkt, and β-actin detected using Western blotting. (**a1**) Representative images of PC3 cells. (**a2**) Quantitative data of phosphorylated Akt in PC3 cells. (**b1**) Representative images of DU145 cells. (**b2**) Quantitative data of phosphorylated Akt in DU145 cells. Data are presented as the mean ± SD; * *p* < 0.05 and ** *p* < 0.01 compared with untreated cells.

Since Akt is a major mediator of cell survival through direct inhibition of pro-apoptotic proteins, we investigated whether the effects of CU 2.5 μg/mL, CU 5 μg/mL, and LF 175 μM, alone or in association, were subject to Akt-dependent regulation. To investigate this, we evaluated the activation status of the Akt signaling pathway in PC3 and DU145 cells by Western blot analysis. As shown in Figure 9a, the phosphorylation of Akt in PC3 cells, after 24 h treatments, was reduced relative to untreated cells by treatment with CU 2.5 μg/mL, LF 175 μM, and the combination of CU 5 μg/mL with LF 175 μM. The treatments of DU145 showed a reduction in Akt phosphorylation relative to untreated cells in all the tested conditions, with a significant reduction in the presence of CU 5 μg/mL alone (*p* = 0.002) and CU 2.5 μg/mL combined with LF (*p* = 0.049) (Figure 9b).

### 3.7. The PPI Network

**Figure 10:**
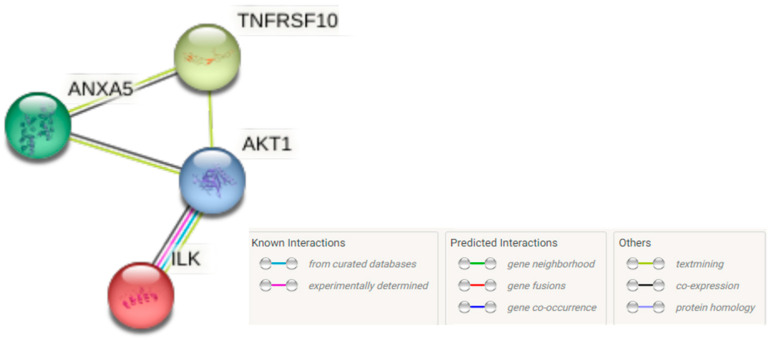
The STRING network connectivity among annexin V, integrin, TRAIL receptors, and the Akt pathway. ILK: Integrin-linked protein kinase; ANXA5: Annexin A5; TNFSF10: Tumor necrosis factor receptor superfamily member; Akt1: RAC-alpha serine/threonine-protein kinase.

Finally, we also evaluated the connectivity, at the protein-interaction level, between the genes analyzed in the DU145 and PC3 prostate cells, modulated by CU and LF. The PPI network involved all the proteins included in the analysis (Figure 10). We observed that our proteins present a PPI among themselves with a *p*-value equal to 0.0137, relative to what would be expected for a random set of proteins of the same size and degree distribution drawn from the genome. Such an enrichment indicates that the proteins are at least partially biologically connected, as a group.

According to the classification for KEGG pathways, the analyzed molecules had a false discovery rate of 0.0465, suggesting a high involvement of the studied molecules in relation to the regulation of the apoptosis pathway; this was also confirmed by the WikiPathway description, in which apoptosis has a 0.0154 false discovery rate with the involvement of the searched molecules.

## 4. Discussion

Cancer treatments are constantly at the center of many debates, due to the high variability of response by cancer cells and by different individuals. To date, prostate cancer treatments have been evaluated through the introduction of targeted and inhibitory therapies toward specific androgen receptors [30]. However, patients show a highly variable response to these therapies, with the concomitant development of drug resistance [31] and the presence of side effects [32]. Much of the interest is aimed at discovering alternative or adjuvant treatments to improve the state of patients suffering from prostate cancer. One of the most-investigated strategies is related to the use of non-toxic natural products [33]. In the present study, the effects of CU and LF, alone and in combination, were examined with respect to prostatic cancer cell proliferation, migration and invasion capability, and pathways involved in apoptosis (the intrinsic annexin V, extrinsic DR expression, and Akt pathway activation).

The anticancer efficacy of CU has been assayed by several studies [34,35,36], but its use in clinical practice is not yet possible due to its reduced bioavailability [37]. Several strategies have been proposed to overcome this limitation and, in this regard, we investigated the use of low doses of CU in combination with LF [38,39]. In the current study, we showed that the cell growth of the two prostatic cell lines analyzed is differently affected by the chosen treatments. In PC3, although there is a cell growth reduction in the presence of all treatments, a more significant reduction was detected in the presence of CU, both at 2.5 μg/mL and 5 μg/mL. Likewise in DU145, we detected a reduction in cell growth, but it was more evident in the presence of LF, either alone or in association with CU. In contrast, we observed that the effect on cell growth is not directly correlated with the effect on the migratory ability of these cells. Our results indicate that in PC3 and DU145 cells, the migratory ability is affected mostly by the combination of CU and LF, which kept the area free of cells more so than in untreated cells or in cells treated with CU or LF alone. Furthermore, this synergistic mixture also reduced the migration of both kinds of prostatic cells as early as 6 h after treatment. These results are supported by previous evidence reporting the high capability of LF [40,41] and CU [42,43] to affect cancer cell migration, both in vivo and in vitro, suggesting their combined use as a possible strategy to inhibit the invasiveness of prostate cancer cells. Thus, to better understand the anti-migratory and anti-invasive effects associated with treatment with CU and LF, we evaluated the expression of integrins, membrane glycoproteins that bind extracellular matrix proteins. Each integrin is made up of two chains. In the context of tumor regulation, an important role is played by the α3 and β1 integrins, involved in tumor progression and responsible for the formation of metastases, increasing the invasiveness of tumor cells [44]. In conditions of homeostatic equilibrium, the integrins determine the induction of cell survival signaling, to prevent cell apoptosis. In our in vitro model of prostate cancer cells, we demonstrated that the combination of CU 2.5 μg/mL and LF 175 μM was significantly related to the down-expression of integrin α3 and, although not significantly, to the β1 subunit gene expression in PC3 and DU145; this supports the possible role of natural products in promoting the maintenance of the homeostatic environment. According to migration assays, LF significantly inhibited migration and invasion capacities and reduced the expression of integrins, mainly the integrin α3 subunit. The different balance between integrin subunit expression and the invasion of tumor cells is dependent on the metastatic potential of PC3 and DU145 [25].

Several studies have described CU as responsible for apoptosis induction in a wide variety of cell lines [45,46], and have suggested that CU treatment promotes an increased level of apoptosis in earlier phases of wound healing [47]. Induction of apoptosis is one of the fundamental mechanisms that impedes cancer growth and proliferation, and LF has been demonstrated to be involved in the apoptosis pathways [48,49], with accumulating evidence supporting the enhancement by LF of DR4 and DR5 expression with sensitization of TRAIL-induced apoptosis [50]. Thus, to evaluate whether CU and LF as a combined treatment inhibited cancer cell growth by induction of apoptosis, we evaluated DR gene expression and annexin V quantification to detect apoptotic pathway regulation. Our results showed that the expression levels of DR4 and DR5 were increased by CU and LF in both DU145 and PC3 cell lines, in accordance with Dai et al. [51].

Curcumin [52] and LF [53] have previously been reported to induce apoptosis in prostate cells, such as PC3 and DU145. In our experimental approach, we found that in PC3 cells, CU alone induces an increase in annexin V levels, although not significantly in a dose-dependent manner; moreover, we found that CU in association with LF leads to a significant increase in annexin V levels. Furthermore, in DU145 cells, the significant increases in annexin V levels are related to individual treatments, suggesting a cell-specific mechanism.

Subsequently, in our in vitro experiments, Akt signaling pathways were shown to be modulated by CU and LF, alone and in combination. Akt, well known as a key survival factor involved in the control of cell proliferation and apoptosis, and described as a therapeutic target used in oncology, shows a reduced activation, supporting CU and LF’s roles as inhibitors of the growth, proliferation, adhesion, and invasion of prostate cancer cells [37].

Overall, our results underline that a low concentration of CU can reduce cell invasion and proliferation, together with the ability to promote the activation of the apoptotic pathway. Moreover, the combination of CU with LF amplifies these effects. Indeed, our data highlight the concomitant ability of CU and LF with respect to the reduction of cell migration, the down-regulation of integrin gene expression, and the up-regulation of DR4 and DR5 gene expression.

## 5. Conclusions

In conclusion, CU and LF, alone and in combination, result in a reduction in the aggressiveness of prostatic cancer cells, mainly through the reduction of apoptotic pathway mediators and cell migratory ability, supporting the beneficial role of CU and LF in cancer therapy and as adjuvant nutrients.

The significant effects obtained from the application of a low dose of CU (2.5 and 5 μg/mL) both with and without LF, represent one of the crucial findings of our work. These novel findings may have important implications for the maintenance of the quality of life of prostate cancer patients. The challenge of the natural product’s utilization and integration in therapy decisions is complex, and includes developing a better understanding of networks and the phenomenon of different tissue models. The identification of how CU and LF, alone or in combination, act on health needs to be assessed in terms of their effectiveness, the evaluation of the potential adverse effects in real-life conditions, and possible sources of pharmacological interference.

## Data Availability

Not applicable.
